# Protein ubiquitination in ovarian cancer immunotherapy: The progress and therapeutic strategy

**DOI:** 10.1016/j.gendis.2023.101158

**Published:** 2023-10-29

**Authors:** Huiling Guo, Jianwei Wei, Yuyan Zhang, Li Wang, Junhu Wan, Weiwei Wang, Ling Gao, Jiajing Li, Ting Sun, Liwei Ma

**Affiliations:** a Department of Clinical Laboratory, The First Affiliated Hospital of Zhengzhou University, Zhengzhou, Henan 450052, China; b Key Clinical Laboratory of Henan Province, Zhengzhou, Henan 450052, China; c Department of Neurosurgery, The First Affiliated Hospital of Zhengzhou University, Zhengzhou, Henan 450052, China; d Department of Pathology, The First Affiliated Hospital of Zhengzhou University, Zhengzhou, Henan 450052, China; e Department of Gynecologic Oncology, The Affiliated Cancer Hospital of Zhengzhou University & Henan Cancer Hospital, Zhengzhou, Henan 450052, China

**Keywords:** Deubiquitination, Immune-related molecules, Immunotherapy, Ovarian cancer, Ubiquitination

## Abstract

Ovarian cancer is a common cancer for females, and the incidence and mortality rates are on the rise. Many treatment strategies have been developed for ovarian cancer, including chemotherapy and immunotherapy, but they are often ineffective and prone to drug resistance. Protein ubiquitination is an important class of post-translation modifications that have been found to be associated with various human diseases and cancer development. Recent studies have revealed that protein ubiquitination is involved in the progression of ovarian cancer and plays an important role in the tumor immune process. Moreover, the combination of ubiquitinase/deubiquitinase inhibitors and cancer immunotherapy approaches can effectively reduce treatment resistance and improve treatment efficacy, which provides new ideas for cancer treatment. Herein, we review the role of protein ubiquitination in relation to ovarian cancer immunotherapy and recent advances in the use of ubiquitinase/deubiquitinase inhibitors in combination with cancer immunotherapy.

## Introduction

Ovarian cancer is a multifrequency female cancer, which became the second leading cause of death from gynecological cancers in China after about 2005, with the incidence and mortality rates of ovarian cancer also increasing in recent years.^1^ In 2020, a total of 313,959 new cases and 207,252 new deaths occurred worldwide, ranking eighth in terms of incidence and mortality of all cancers.^2^ Although there are new treatments available for ovarian cancer, treatment outcomes and overall survival rates for ovarian cancer have not improved because more than 70% of ovarian cancers are not diagnosed until they reach stage III or IV, and most patients experience recurrence and chemo-resistance after chemotherapy. Currently, surgery and chemotherapy are the main treatments for ovarian cancer, however, patients often experience chemo-resistant relapse within a few years after the initial treatment, so various immunotherapies are being investigated for adjuvant treatment, including immune checkpoint inhibition, adoptive T cell-receptor therapy, and intraperitoneal monocytes plus type I interferons (IFNs) as a cellular immunotherapy.^3^, ^4^, ^5^, ^6^, ^7^ While immunotherapy has made great progress in recent years, ovarian cancer has a limited response to immunotherapy.^8^^,^^9^

Ubiquitin-protein conjugation is a type of protein hydrolysis-dependent or non-dependent post-transcriptional modification. Ubiquitination is catalyzed by a three-enzyme cascade reaction consisting of E1 (ubiquitin-activating enzyme), E2 (ubiquitin-conjugating enzymes), and E3 (ubiquitin-ligase enzymes), with E3 ubiquitin-ligase enzymes playing a key role in regulating the cascade of ubiquitin transfer reactions by recognizing and catalyzing the coupling of ubiquitin to specific protein substrates.^10^^,^^11^ Ubiquitination is a dynamic and reversible process, the deubiquitination is catalyzed by deubiquitinating enzymes (DUBs) that perform the function of removing protein ubiquitination.^11^ The ubiquitination/deubiquitination process plays an important role in different aspects of the organism. On one hand, it is involved in regulating many aspects of the immune system, including the development, activation, and differentiation of lymphocytes, induction of T-cell tolerance, antigen presentation, immune evasion, and viral outgrowth. On the other hand, in addition to proteasome-mediated degradation, E3-promoted ubiquitination affects a wide range of biological processes, such as receptor down-regulation, signal transduction, protein processing or translocation, protein–protein interactions, and gene transcription.^10^ Ubiquitination/deubiquitination-mediated protein degradation plays an important role in cell cycle progression, signal transduction, transcriptional regulation, receptor down-regulation, and cytokinesis, among other processes.^12^ Several studies in recent years have shown that the process of ubiquitination is closely associated with the progression of ovarian cancer and chemotherapy resistance. F-box proteins can recruit substrates through protein–protein interactions and promote substrate ubiquitination and degradation. The F-box protein (FBP) family of E3 ubiquitin ligases such as FBXO2 and FBXO6 can contribute to the development of ovarian cancer by performing their ubiquitin ligase functions to promote the ubiquitinated degradation of their substrates. Other E3 ubiquitin ligases, like cullin 3, also promote ovarian cancer development through the ubiquitinated degradation of their substrates.^13^, ^14^, ^15^, ^16^ In addition, there are also E3 ubiquitin ligases that can inhibit the development of ovarian cancer, such as FBXO16, which also depends on the degradation of the substrate by its ubiquitin ligase activity.^17^ Also, deubiquitination plays a significant role in ovarian carcinogenesis, for example, deubiquitinase PSMD14 is highly expressed in ovarian cancer and promotes ovarian cancer progression by reducing the ubiquitination of substrates.^18^

In conclusion, ubiquitination/deubiquitination has an important effect on ovarian cancer. It was also found that ubiquitination was associated with the efficacy of chemotherapy and immunotherapy in ovarian cancer. During the process of immunotherapy, ubiquitination/deubiquitination is prone to cause a resistance to immunotherapy thus influencing the efficacy of the treatment.^19^^,^^20^ Moreover, the ubiquitination process has been found to influence chemoresistance in ovarian cancer, and E3 ubiquitin ligases have been shown to play a key role in chemoresistance by degrading various chemoresistance-associated substrates in ovarian cancer.^21^, ^22^, ^23^, ^24^, ^25^

### Ubiquitination/deubiquitination process

Ubiquitin is a protein consisting of 76 amino acids.^26^ Ubiquitination is catalyzed by three enzymes (E1, E2, and E3); ubiquitin is first activated by E1, the activated ubiquitin binds to E2, and subsequently, E2 transfers the activated ubiquitin to the target protein recognized by E3.^27^, ^28^, ^29^ In most cases, the isopeptide bond is formed between the internal Lys (K) wε-amino of the substrate protein and the ubiquitin c-terminal carboxyl group.^29^ The key feature of ubiquitin is its seven Lys residues (K6, K11, K27, K33, K48, and K63), all of which can be ubiquitinated to produce an isopeptide-linked ubiquitin chain. The eighth chain type, Met1-linked or “linear” chain, is created when the ubiquitin is connected to the N-terminal end of the second ubiquitin.^26^ Among them, K48 and K63 are the most abundantly studied, but nowadays the study of ubiquitination of other species is gradually deepening.^30^

**Figure 1:**
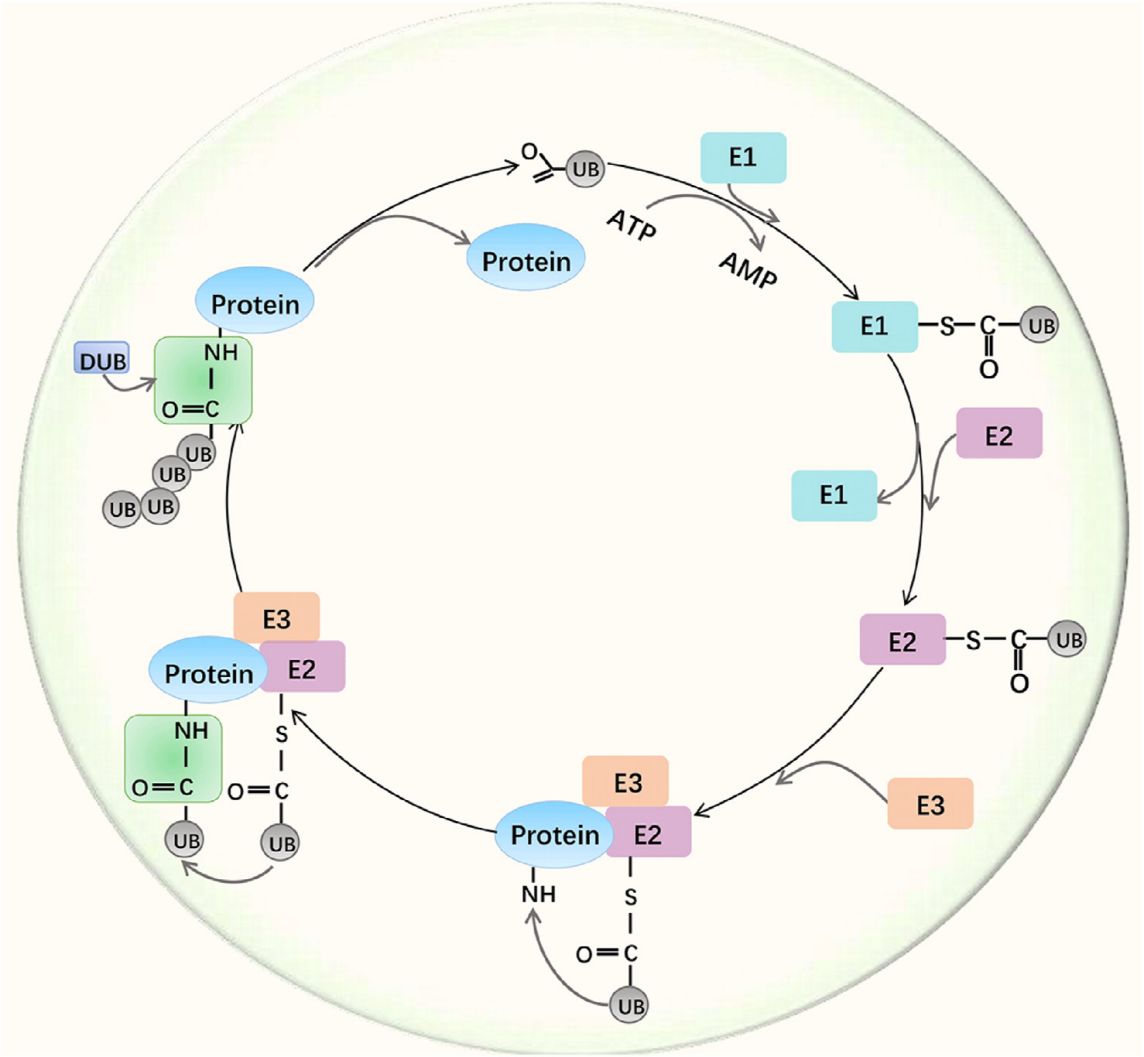
The process of ubiquitination/deubiquitination.

The deubiquitination process is catalyzed by DUBs, reversing the degradation caused by ubiquitination on the one hand and other functional changes caused by ubiquitination on the other hand.^31^ Depending on the different catalytic mechanisms, there are nearly one hundred known DUBs, including cysteine proteases (USPs, UCHs, MJDs, and OTOs) and metalloproteases (containing metallo-catalytic structural domains).^32^ DUBs are not only involved in the recycling and conversion of ubiquitinated ubiquitin, but they rearrange ubiquitin-linked proteins.^33^ DUBs exert deubiquitination by hydrolyzing the isopeptide bond between ubiquitin and the target protein, and all DUBs have at least one ubiquitin-binding site, such as the S1 site. S1 site selection for ubiquitination-modified proteins to deubiquitinate the ubiquitin C-terminus and scissor bond-guided active site.^34^ In cleavage of ubiquitin, the S1 site is occupied by distal ubiquitin while proximal ubiquitin occupies the S1 site, and besides that, some other DUBs have additional ubiquitin binding sites, such as S2 and S3 (Fig. 1).^35^

## Ubiquitination/deubiquitination and tumor immune

### Tumor microenvironment

In the process of ovarian cancer immunotherapy, drug resistance often occurs, which affects the survival and prognosis of patients. Meanwhile, the tumor microenvironment contains a variety of immune cells, which is closely related to the immunotherapy effects. Also, many components of the tumor microenvironment are regulated by ubiquitination/deubiquitination.^36^

Macrophages play an important role in antitumor immunity and can be usually divided into classically activated M1 macrophages and selectively activated M2 macrophages. Tumor-associated macrophages, usually presenting as M2, act as inhibitors of the cytotoxic function of tumor-killing immune cells, thus weakening anti-tumor immunity and thereby contributing to adverse tumor outcomes as well as increasing the difficulty of treatment.^37^ Ubiquitination plays a prominent role in regulating macrophages, and the IGF2BP3 (insulin-like growth factor 2 mRNA-binding protein 3) protein, a known oncogenic protein promotes immunosuppressive phenotypic polarization in macrophages.^38^ The stability of IGF2BP3 can be down-regulated by E3 ubiquitin ligases HECTD4 and TRIM25-mediated ubiquitination to regulate tumor-associated macrophage infiltration.^38^^,^^39^ A recent study found that the E3 ubiquitinase UBR5 is critical for ovarian cancer progression as it promotes the recruitment and activation of tumor-associated macrophages through key chemokines and cytokines.^21^ Also, there was a strong relationship between the expression level of the E3 ubiquitin ligase MEX3A and the infiltration level of macrophages, neutrophils, dendritic cells, B cells, and CD8^+^ T cells.^40^ In addition to ubiquitination, deubiquitination can also regulate the progression of tumors and tumor-associated macrophage polarization. USP10 mediates the deubiquitination of NLRP3 to enhance its protein stability, which promotes the secretion of NLRP3-induced C–C motif chemokine ligand 2 and promotes the polarization of pro-tumorigenic M2-like macrophages in colorectal cancer.^41^

Regulatory T cells (Tregs) are necessary for the control of the immune response and the maintenance of homeostasis in the body while impeding anti-tumor immunotherapy. Several studies have also shown that ubiquitination/deubiquitination processes play an important role in the regulation of Treg. Treg exhibits instability when it loses the transcription factor Foxp3, which can promote tumor immunity, and a variety of ubiquitinases/deubiquitinases can regulate Treg by modulating Foxp3. E3 ubiquitin ligase Rnf20 inhibits Treg by promoting histone ubiquitination on the Foxp3 promoter and conserved non-coding DNA sequence regions leading to chromatin condensation and Foxp3 transcriptional deletion.^42^^,^^43^ In contrast, deubiquitinases alleviate ubiquitinase-induced Treg instability, and Usp22 and Usp21 maintain Foxp3 expression at the transcriptional and post-translational levels through DUB function. Hence, they regulate the metabolic program of Treg cells and promote the adaptation of Treg cells in nutrient-limited environments.^43^

Cancer-associated fibroblasts (CAFs) are the main cellular components that form the tumor microenvironment and are closely related to tumor growth and metastasis.^44^^,^^45^ Similar to macrophages and Treg, ubiquitination/deubiquitination also acts on CAFs to regulate tumor progression. Ubiquitination/deubiquitination prevents the activation of CAFs by targeting PRKN to inhibit mitochondrial autophagy, which in turn inhibits proline synthesis and induces defective autophagy in CAFs.^45^ In addition to this, ubiquitination/deubiquitination also plays a part in downstream of CAF. usp7 promotes the entry of CAF-derived miR522 into the exosome by stabilizing hnRNPA1 through deubiquitination, which regulates tumor progression through the usp7/hnRNPA1 axis.^46^ Thus, ubiquitination/deubiquitination can be involved in tumor progression by modulating CAFs or participating in CAF downstream pathways.

### Tumor immune responses

A critical mechanism of cancer immune escape is defective processing and presentation of tumor antigens, including loss of major histocompatibility (MHC) expression or dysregulation of antigen processing mechanisms.^47^ MHC I and MHC II play a key role in the activation and regulation of adaptive immunity by presenting antigens to CD8^+^ or CD4^+^ T cells.^48^^,^^49^ Moreover, the ubiquitin-proteasome system has been shown to influence the process of antigen presentation by regulating the expression of MHC Ⅰ and MHC Ⅱ, thus affecting the immune process. Ubiquitin-proteasome system is widely recognized to play an important role in MHC Ⅰ regulation. A recent study estimated its contribution to MHC class I peptide production by quantitative mass spectrometry. The results revealed that ubiquitin-proteasome system is required for the production of most MHC class I peptide complexes and demonstrated in breast cancer cells that proteasome inhibitors have potential for use in cancer immunotherapy.^50^ First, ubiquitinase binds directly to MHC Ⅰ, ubiquitinates and degrades MHC I, and thus inhibits the expression of MHC Ⅰ. MHC Ⅰ binds to the surface protein sushi structural domain 6 and transmembrane protein 127 to form a three-molecule complex, which recruits the E3 ubiquitin ligase WWP2. Then WWP2 activates the ubiquitination and lysosomal degradation of MHC Ⅰ, leading to an effect on the antigen presentation process.^51^ Furthermore, ubiquitination can also indirectly regulate the expression of MHC Ⅰ. E3 ubiquitin ligase VHL can induce the activation of the JAK1/STAT1 pathway by degrading PTP1B and TC-PTP, thus promoting the expression of MCH Ⅰ in tumor cells.^48^ The increased expression of MHC Ⅰ contributes to increasing IFN-γ signaling and antigen presentation in tumor cells as well as promoting CD8^+^ T cell activation.^48^ In the case of MHC Ⅱ, ubiquitination can regulate the expression of MHC Ⅱ at the transcriptional level. The E3 ubiquitin ligase FBXO11 regulates MHC class II transactivator protein levels through ubiquitination-mediated degradation. MHC class II transactivator, a major regulator of MHC Ⅱ gene transcription, leads to reduced MHC II at the transcriptional level after degradation by the ubiquitinated proteasome. Thus, the E3 ubiquitin ligase FBXO11 is negatively associated with MHC Ⅱ.^49^ It is also known that MHC Ⅱ can be directly ubiquitinated and degraded by the E3 ubiquitin ligase MARCH1 and thus affects the antigen presentation of dendritic cells as well as humoral immunity.^52^ Interestingly, ubiquitination of MHC Ⅱ has also been found to influence the expression of MHC Ⅰ. The expression of MHC I on the surface can be significantly reduced by inhibiting MHC II ubiquitination, but the specific mechanism has not been fully clarified; it is speculated that MHC II and MHC I may compete for the recycling pathway, or excessive MHC II molecules disturb the microstructural domains, which makes MHC I easy to be destabilized.^53^ In conclusion, ubiquitination is closely associated with the expression of MHC Ⅰ and MHC Ⅱ. As MHC Ⅰ and Ⅱ mediate antigen presentation, they correlate with the efficacy of tumor immunotherapy such as checkpoint immune blockade therapy and anti-tumor antibody immunotherapy.^49^ Thus targeting ubiquitination and antigen presentation may help to improve the efficacy of tumor immunotherapy.

In the tumor microenvironment, antigen-presenting cells recognize tumor-derived DNA thereby driving STING signaling activation. Activation of STING signaling promotes the generation of an immune response and the activation of a T-cell-dependent anti-tumor immune response. This plays a crucial role in triggering multiple anti-tumor immune responses.^54^ Ubiquitination can effectively regulate this immune signaling process. The E3 ubiquitin ligase LOM7 can directly interact with STING to promote k63-linked polyubiquitination, thereby inhibiting STING signaling.^54^ Moreover, the ubiquitinases TRIM24 and TRIM25 can also affect STING signaling by regulating DNA signaling. TRIM25 promotes the ubiquitination and degradation of mitochondrial voltage-dependent anion-selective channel protein 2, which inhibits mitochondrial DNA released from nasopharyngeal carcinoma radiotherapy to suppress the type I interferon response produced after radiotherapy.^55^ TRIM24 induces the degradation of TREX1. Accumulation of cytoplasmic DNA induced by TREX1 degradation activates the cytoplasmic DNA sensing cGAS/STING pathway, leading to induction of type I interferon.^56^ The above ubiquitination process regulates STING signaling, which can further affect the activation of type I interferon under STING signaling, thereby influencing the inflammatory response in the tumor microenvironment.^54^, ^55^, ^56^ In addition, the E3 ubiquitin ligase STUB1 can directly mediate the ubiquitin-dependent proteasomal degradation of the IFNγ-R1/JAK1 complex, thereby inhibiting interferon-mediated inflammatory responses.^57^

### Ubiquitination/deubiquitination and ovarian cancer immunotherapy

**Figure 2:**
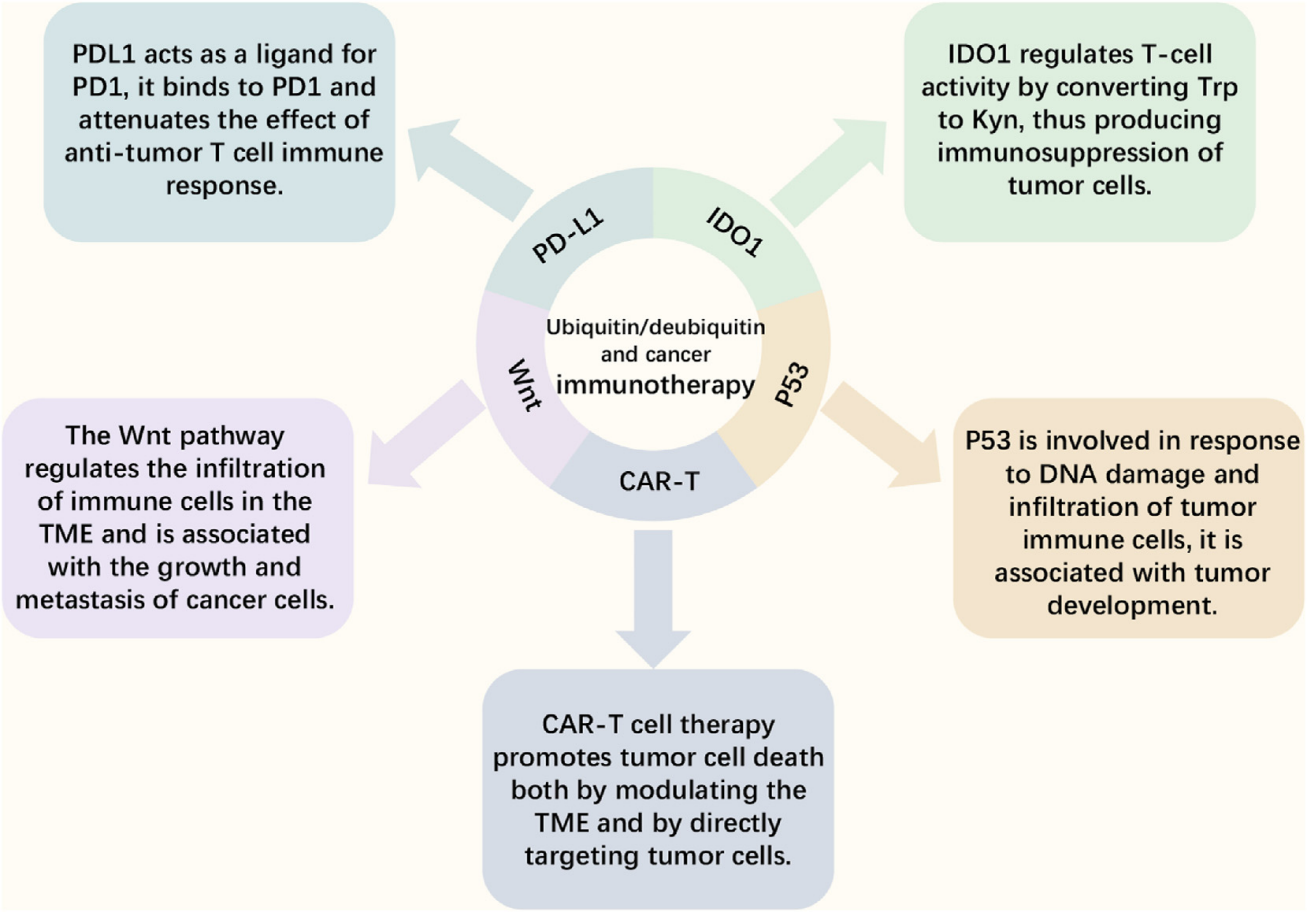
Main mechanisms of cancer immunotherapy that can be modulated by ubiquitination/deubiquitination. CAR, chimeric antigen receptor; Kyn, kynurenine; IDO1, indoleamine 2,3-dioxygenase 1; PD-1, programmed cell death-1; PD-L1, programmed cell death-ligand 1; TME, tumor microenvironment; Trp, tryptophan.

Ubiquitination/deubiquitination processes play an important role in the repair of DNA damage, regulation of the cell cycle, and immune response.^58^, ^59^, ^60^ Several immunotherapies have been identified for ovarian cancer treatment (Fig. 2) but with limited efficacy, and researchers have found that ubiquitination/deubiquitination can regulate ovarian cancer development in several ways and is associated with immunotherapy for ovarian cancer.^8^^,^^61^ Several immunotherapy-specific targets of ubiquitination/deubiquitination modifications are as follows (Table 1).

**Table 1:** Several specific immune-related targets of ubiquitination/deubiquitination modification.

Molecules	Enzymes	Ubiquitin chain	Modification sites	Cancer types	Functions	Reference
PD-L1	β-TRCP	__	__	Ovarian cancer	It promotes intracellular PD-L1 ubiquitinated proteasome degradation, enabling PD-L1 treatment-insensitive tumor cells to become sensitive and promoting immune checkpoint blockade treatment.	73
	USP7	__	__	Gastric cancer	USP7 deubiquitinates PD-L1 as a deubiquitinating enzyme, which stabilizes PD-L1 and inhibits anti-tumor immunity.	70
	FBXO22	__	__	Lung adenocarcinoma	FBXO22 ubiquitinates and degrades PD-L1 through proteasome-mediated degradation, and sensitizes cancer cells to DNA damage.	71
IDO1	USP14	K48	__	Colorectal cancer	It deubiquitinates and stabilizes IDO1 to prevent the degradation of IDO1 by TRIM21, thus promoting tryptophan metabolism and immunosuppression.	81
	TRIM21	K48	__	Colorectal cancer	It ubiquitinated and degraded IDO1, inhibiting IDO1-mediated tryptophan metabolism and immunosuppression.	81
p53	RBX1	__	__	Ovarian cancer	It increases the proliferation and decreases the apoptosis of tumor cells by enhancing ubiquitination and proteasome degradation of p53.	95
	Parkin	__	__	Ovarian cancer	It mediates p53 ubiquitination and proteasome degradation that inhibits the proliferation of ovarian cancer cells.	96
	SMYD3，UBE2R2	__	__	Epithelial ovarian cancer	SMYD3 promotes the transfer of p53 from the nucleus to the cytoplasm and interacts with UBE2R2 to promote the ubiquitinated degradation of p53 which enhances the migration ability of epithelial ovarian cancer cells.	97
mtp53	TRIM27	K11, K27, K29, K63	__	Ovarian cancer	It promotes the ubiquitination and degradation of mtp53, thereby inhibiting the proliferation and invasion of ovarian cancer cells.	99
	DTX3/RNF154	K11, K27, K29, K33, K48, K63	__	Ovarian cancer	It inhibits the binding of MDM2 to p53 by ubiquitinating mtp53, resulting in the stabilization of p53 and promoting the growth and proliferation of ovarian cancer cells.	98
mtp53-R175	USP15	__	__	Ovarian cancer	Loss of USP causes the ubiquitination of p53-R175 and the degradation by lysosomes, which reduces the viability of cancer cells.	100
CAR	K3/K5	__	__	__	It mediates ubiquitinated degradation of MHC I/II on CAR-T to evade the host immune response and maintain the survival of CAR-T cells	111
Frizzled (FZD)	RNF43	__	__	Gastric cancer	RNF43 mediates ubiquitination and lysosomal degradation of FZD, thereby inhibiting Wnt/β-catenin signaling pathway activation.	125
c-MYC protein	TRIM37, HUWEI	__	__	Ovarian cancer	TRIM37 promotes the expression of c-MYC by targeting HUWE1.	139
β-catenin	UBE2S, APC/C	K11	K19	High-grade plasma ovarian cancer	UBE2S interacts with the APC/C complex to ubiquitinate the K19 residue of β-catenin and improve its stability, thereby preventing its degradation and activating the Wnt/β-catenin signaling pathway.	134,135
	UCHL5	__	__	Endometrial cancer	UCHL5 inhibits ubiquitinated proteasome degradation of β-catenin and activates the Wnt/β-catenin signaling pathway.	136

### PD-L1

**Figure 3:**
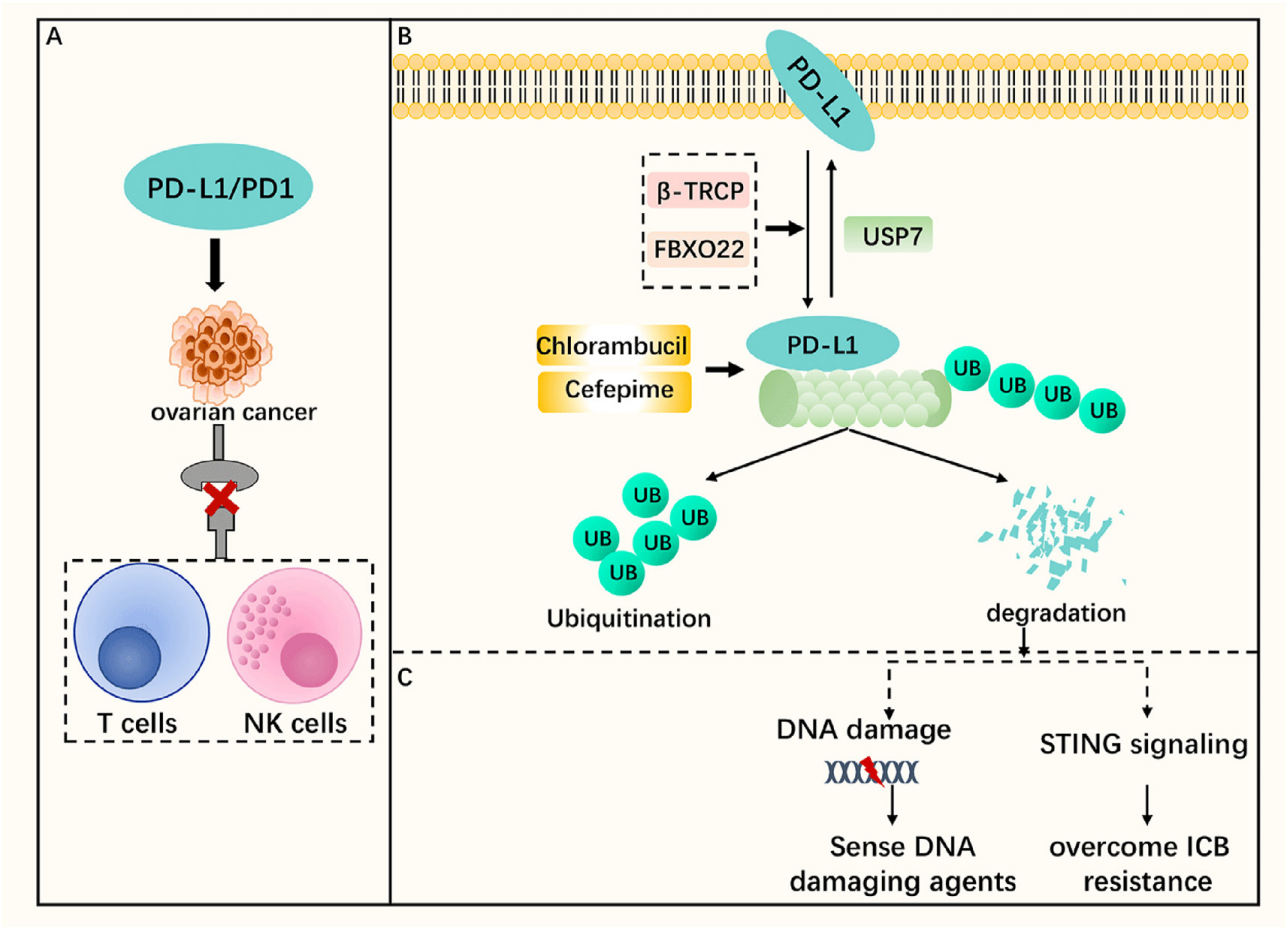
PD-L1 with ubiquitination/deubiquitination. **(A)** PD-L1/PD-1 can block the killing of tumor cells by T cells and natural killer (NK) cells. **(B)** The ubiquitinase β-TRCP and FBXO22 induce PD-L1 ubiquitination and proteasomal degradation, while the PD-L1-depleting drugs cefepime and chlorambucil promote PD-L1 ubiquitination and proteasomal degradation in ovarian cancer cell lines. The deubiquitinase USP7 removes the ubiquitination of PD-L1. **(C)** Ubiquitinated degradation of PD-L1 inhibits the repair of DNA damage, makes cells more sensitive to DNA damage, and activates STING signaling to overcome immune checkpoint blockade (ICB) resistance. PD-1, programmed death-1; PD-L1, programmed death-ligand 1; UB, ubiquitin.

Programmed death-ligand 1 (PD-L1, CD274) is a B7 homologous family immune co-signaling molecule that interacts with programmed death-1 (PD-1), a receptor on T cells and natural killer cells, to inactivate both T and natural killer cells, thus attenuate anti-tumor immune responses and allow tumors to evade immune surveillance (Fig. 3A).^62^, ^63^, ^64^ Drugs that block PD-1 or PD-L1 promote endogenous anti-tumor immunity and have been considered the common standard of care for cancer treatment due to their broad spectrum of activity.^62^ In addition, PD-L1 protects tumor cells from the cytotoxic effects of type I and type II interferons and cytotoxic T lymphocyte-mediated cell lysis, which is a process that does not require PD-1 signaling in T cells.^65^ Therefore, PD-L1 is an effective target for regulating tumor immunity. Anti-PD-L1 antibodies are thought to play an important role in the adjuvant treatment of ovarian cancer,^66^ and PD-L1 may be a prognostic indicator for ovarian cancer.^67^ Recently, it has been shown that the regulation of PD-L1 includes degradation through ubiquitination,^20^ suggesting that ubiquitination is involved in PD-L1 immunotherapy. Immune checkpoint blockade (ICB) is well known and studied for its ability to counteract PD-L1 expressed on the cell surface, but recent evidence on cell-intrinsic PD-L1 signaling in immunopathogenic tumors suggests an additional role for cell-intrinsic PD-L1 beyond its typical surface role. These roles are important in treatment resistance, where in particular they are in the resistance of oncology treatments to different classes of therapies, including cytotoxic drugs, targeted small molecules, radiation, and immunotherapy.^68^

Murine double minute 2 (MDM2), an E3 ubiquitin ligase, is highly expressed in ovarian clear carcinoma cells, and MDM2 was found to inhibit T cell-mediated tumor killing induced by ICB; AMG232 is a selective MDM2 inhibitor. The combination of AMG232 and anti-PD-L1 therapy can increase the ability of T cells to kill tumors and overcome drug resistance, suggesting that the potential of E3 ubiquitin ligase as a target to participate in PD-L1 ICB therapy, but the exact mechanism is not yet clear and further *in vivo* experiments are needed for its verification.^69^ In the course of studies on other types of tumors, ubiquitinating and deubiquitinating enzymes have been found to regulate PD-L1 expression, for example, the deubiquitinating enzyme USP7 removes PD-L1 ubiquitination in gastric cancer cells^70^ and stabilizes PD-L1; the E3 ligase FBXO22 ubiquitinates and proteasomal degrades PD-L1.^71^ Therefore, the role of ubiquitination and deubiquitination in the regulation of PD-L1 in ovarian cancer still holds a lot of room for exploration. In addition to the classical cancer therapeutic function of surface PD-L1, cell-intrinsic PD-L1 in tumor cells has also been shown to be involved in tumor immunity and regulated by ubiquitination. FDA-approved PD-L1-depleting drugs such as cefepime^72^ and chlorambucil^73^ promote ubiquitination and proteasomal degradation of PD-L1 in ovarian cancer cell lines to regulate PD-L1 expression, and chlorambucil relatively selectively degrades PD-L1 from tumor cells rather than stromal or local immune PD-L1 degradation by ubiquitination in cells.^73^ Chlorambucil has been found to promote intra-cellular PD-L1 ubiquitination via the E3 ligase β-TRCP and GSK3β/β-TRCP-mediated degradation of the PD-L1 proteasome.^73^ However, the specific mechanism by which cefepime promotes ubiquitinated degradation of PD-L1 has not been shown,^72^ and *in vivo* studies are needed for further validation. Chloramphenicol and cefepime reduce DNA damage repair by inhibiting cell-intrinsic PD-L1, and the increased DNA damage activates the immunogenic STING pathway.^72^^,^^73^ STING pathway activation was found to induce cell surface PD-L1 expression, which provides favorable conditions for ICB treatment.^74^ In addition, chloramphenicol increased the expression of natural killer cells in the tumor immune microenvironment and improved anti-tumor immunity, which is beneficial for ICB therapy, but this area still requires further research.^73^

In conclusion, ubiquitination/deubiquitination can regulate the expression of intrinsic and classical PD-L1, thus affecting tumor immunotherapy (including ICB), but its efficacy *in vivo* and specific mechanism have not been proved yet (Fig. 3B). Therefore, the combination of ubiquitination/deubiquitination and PD-L1 for tumor immunotherapy has great research potential and significance.

### IDO1

**Figure 4:**
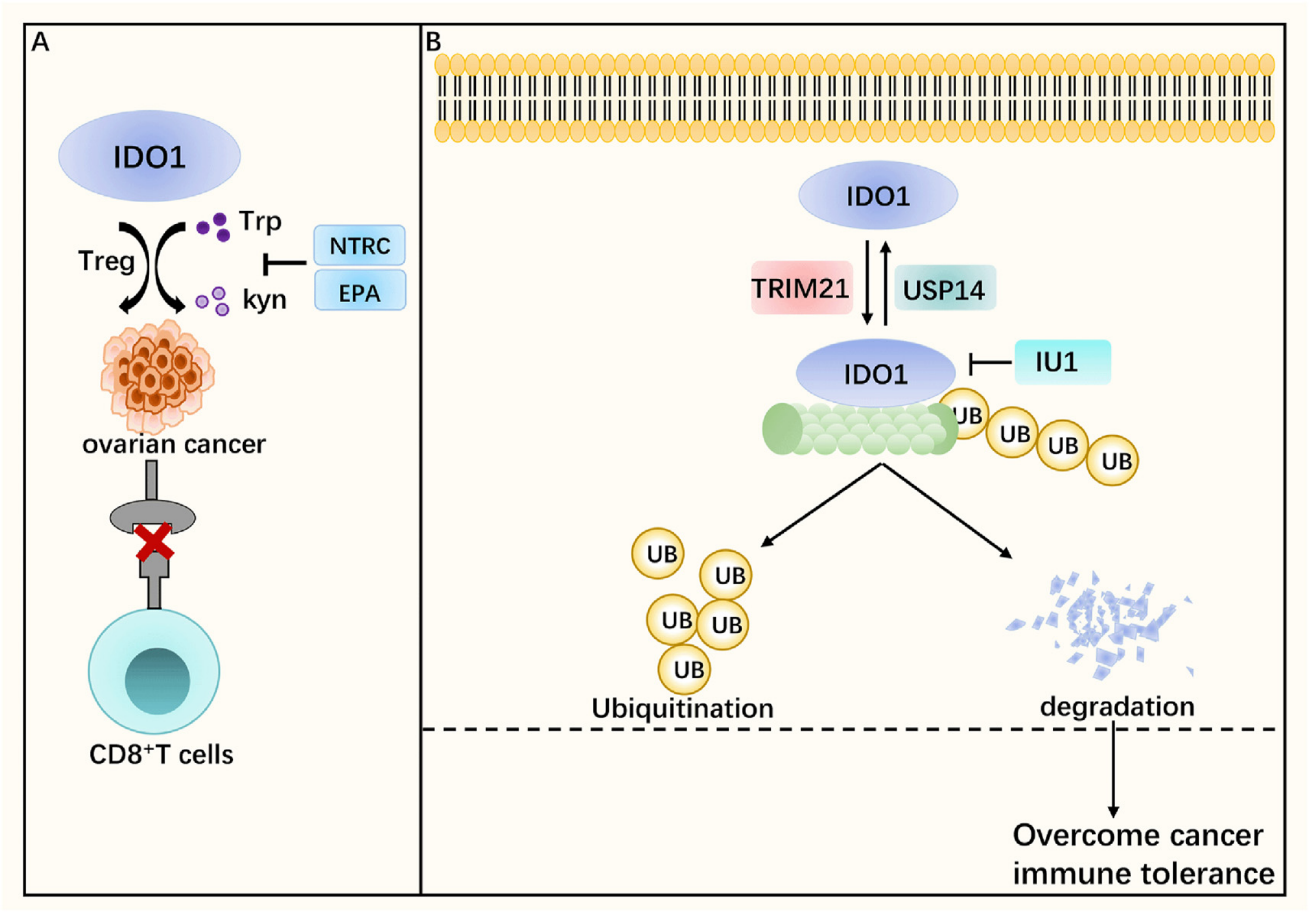
IDO1 with ubiquitination/deubiquitination. **(A)** Basic function of IDO1. IDO1 promotes the conversion of tryptophan (Trp) to kynurenine (Kyn), which up-regulates immunosuppressive Treg and inhibits the activity of CD8^+^ T cells thereby suppressing tumor cell killing by the immune system. NTRC (NTRC3883-0) and EPA (epacadostat) can inhibit IDO1-promoted conversion of Trp to Kyn. **(B)** The process of ubiquitination and deubiquitination of IDO1. The ubiquitinase TRIM21 can mediate the ubiquitination of IDO1 as well as proteasomal degradation. The deubiquitinase USP14 deubiquitinates IDO1 to reduce its degradation, and the IU1, an inhibitor of USP, inhibits the deubiquitination of IDO1. Decreased levels of IDO1 can reverse the immune tolerance of tumors. IDO1, indoleamine 2,3-dioxygenase 1.

Indoleamine 2,3-dioxygenase 1 (IDO1) is a heme-containing enzyme that catalyzes the breakdown of tryptophan (trp), which is essential for immune function, to kynurenine (kyn), thereby inhibiting CD8^+^ T cell activity and up-regulating immunosuppressive Treg, resulting in the inability of the immune system to respond appropriately to cancer cells, and thus IDO1 is considered an important target for cancer immunotherapy (Fig. 4A).^75^ Similar to PD-1/PD-L1, cancer cells have been found to evade the host immune response by inhibiting immune surveillance through the IDO pathway.^76^^,^^77^ IDO1 has now been shown to be highly expressed in ovarian cancer, which is strongly correlated with the infiltration of immune cell populations, especially dendritic cells and T cells, being an important immune-related gene in ovarian cancer.^78^

Various IDO1 inhibitors have been found to improve IDO1-mediated immunosuppression of tumor cells by inhibiting the IDO1 pathway, including NTRC 3883-0 and epacadostat (INCB024360, EPA). Among them, although NTRC 3883-0 can inhibit IDO1, the acceptable dose for cancer patients makes clinical use less meaningful.^79^ EPA is an oral reversible competitive IDO1 inhibitor, but studies found that its treatment as an IDO1 inhibitor was not successful, which seemed to eliminate enthusiasm for IDO1 research, however, subsequent studies identified the problem. The inhibition of IDO1 leads to metabolic adaptation that promotes the NAD^+^ biosynthetic pathway, which inhibits T cell proliferation and expression, and A2a and/or A2b purinergic receptor antagonists were found to block NAD^+^ inhibition of T cells, thereby ameliorating the metabolic adaptation induced by IDO1 inhibition and promoting tumor immunity.^80^ In general, although IDO1 inhibition can be improved by the combination of drugs, there are still many limitations in the use of IDO1 inhibitors, so it is necessary to explore new methods of IDO1 inhibition to lift tumor immunosuppression.

A new study has shown that ubiquitination/de-ubiquitination has a regulatory effect on IDO1 and does not cause AHR activation which leads to treatment ineffectiveness. The ubiquitinating enzyme TRIM21 mediates IDO1 ubiquitination and proteasomal degradation, while the deubiquitinating enzyme USP14 deubiquitinates IDO1 to stabilize it and prevent it from being degraded by TRIM. The ubiquitination/deubiquitination process of IDO1 is mediated by the K48-linked ubiquitin chain, and experiments have shown that the USP inhibitor IU1 can effectively inhibit IDO1 deubiquitination and reduce IDO1 levels, thereby reversing tumor immune tolerance and making tumor cells more sensitive to anti-PD-1.^81^ In conclusion, ubiquitination/deubiquitination as one of the mechanisms regulating post-translation modifications of IDO1 holds promise as a therapeutic target for cancer and has potential for combination therapy with ICB, but further *in vivo* experiments are needed for its validation (Fig. 4B).

### p53

**Figure 5:**
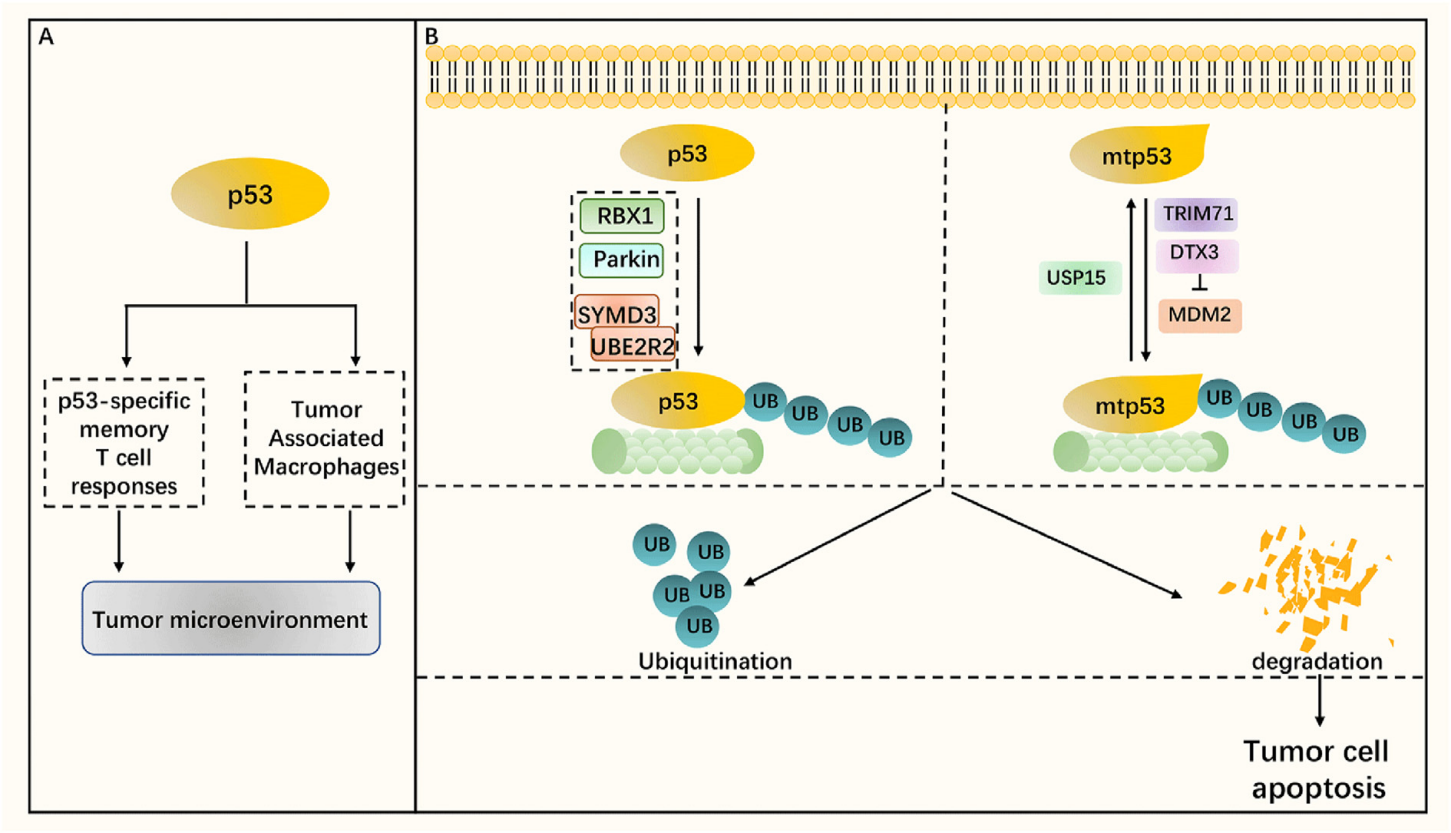
p53/mtp53 with ubiquitination/deubiquitination. **(A)** p53 and tumor microenvironment. p53 can regulate the tumor microenvironment by inducing p53-specific memory T-cell responses and infiltration of tumor-associated macrophages. **(B)** A variety of ubiquitinases can catalyze the ubiquitination and proteasomal degradation of p53, including RBX1, Parkin, SYMD3, and UBE2R2. TRIM71 promotes the ubiquitination and proteasomal degradation of mtp53, while the ubiquitinase DTX3 antagonizes the MDM2-mediated ubiquitinated degradation of mtp53 by ubiquitinating mtp53. The deubiquitinase USP15 then mediates the deubiquitination of mtp53. The ubiquitination/de-ubiquitination process of p53/mtp53 controls tumor cell apoptosis by regulating the expression of p53/mtp53.

p53 protein is encoded by TP53 gene located on chromosome 17p13.1 and is essential for the normal course of the cell cycle. One of the main functions of p53 protein is to activate the transcription of genes that initiate apoptosis in response to DNA damage, and this function is involved in its antitumor activity.^82^^,^^83^ Overexpression of p53 protein has been found in 50%–60% of ovarian cancers, which is associated with mutations in p53. 96% of high-grade serous ovarian cancer cases have p53 mutations, while clear cell ovarian cancer and endometrioid ovarian cancer usually do not carry TP53 mutations.^84^, ^85^, ^86^ p53 plays an important role in the development of ovarian cancer and its immunotherapy. Serum concentrations of anti-p53 autoantibody complexes have been found to be significantly increased in ovarian cancer patients and play an important role in the development of plasma ovarian cancer, as well as affecting survival rates.^87^^,^^88^ Meanwhile, p53 was found to be associated with tumor immune cell infiltration, including induction of p53-specific memory T cell responses with the production of cytokines^89^ and promotion of infiltration by tumor-associated macrophages,^90^ thereby regulating the tumor microenvironment, which makes p53 a possible target for ovarian cancer immunotherapy (Fig. 5A). The use of p53 to construct tumor vaccines has been extensively studied, and several studies to date have shown that p53 vaccines (including p53 synthetic long peptide vaccine, p53MVA vaccine) can induce specific T cell responses by APC treatment, resulting in increased frequency of CD4^+^ T cells, CD8^+^ T cells, and decreased Treg levels. In which the p53MAV vaccine drives an increase in the number of PD-1^+^ CD8^+^ T cells in cancer patients, which increases the likelihood of enhanced immunotherapy efficacy in combination with PD-1/PD-L1.^91^, ^92^, ^93^, ^94^

Ubiquitination and deubiquitination have been found to regulate the expression of p53, both wild-type p53 and mutant p53, with selective specificity for ubiquitination of mtp53. Firstly, several studies on ubiquitination/ubiquitination of wild-type p53 revealed that ubiquitinase/deubiquitinase can regulate the expression of wild-type p53, thus regulating the proliferation and metastasis of tumor cells. E3 ubiquitinase RBX1 can interact with p53 and mediate the ubiquitinated degradation of p53, which inhibits the oncogenic function of p53, thereby increasing tumor cell proliferation and reducing their apoptosis.^95^ Similarly, the E3 enzyme Parkin can increase the ubiquitinated degradation of p53 and inhibit the growth of ovarian cancer cells; moreover, this process can be promoted by metformin, which provides a new mechanism for the treatment of ovarian cancer with metformin.^96^ It was found that SYMD3 interacts with the E2 enzyme UBE2R2 to promote the ubiquitinated degradation of p53, thereby promoting the metastasis of ovarian cancer and enhancing the migration of epithelial ovarian cancer cells, hypothesizing that SYMD3 may have a similar function to that of E3 enzymes.^97^ Secondly, the ubiquitination and deubiquitination of mutant p53 were also investigated. There are many p53 variants in ovarian cancer, so the regulation of mutant p53 is also very important. E3 enzyme DTX3 (also known as RNF154) mediates ubiquitination of mtp53 and maintains stability of mtp53 by preventing MDM2-mediated ubiquitinated degradation of mtp53, thus exerting the pro-oncogenic effect of mtp53 to promote the growth and proliferation of ovarian cancer cells.^98^ Unlike DTX3, which stabilizes mtp53, TRIM71 promotes ubiquitination of mtp53 and proteasomal degradation to destabilize mtp53, thereby inhibiting the growth and invasion of ovarian cancer cells.^99^ It was found that ubiquitinase and deubiquitinase selectively bind to different mutation types of p53 and mediate ubiquitination and deubiquitinase, for example, the deubiquitinase USP15 specifically modifies the P53-R175H mutation type,^100^ but the ubiquitinase MDM2 can regulate the p53-R248Q mutation type.^24^ This strategy of selectively regulating oncogenic mtp53 protein provides ideas for personalized treatment of cancer patients, which can correspond to the type of p53 mutation.

In conclusion, p53, as a proven immunotherapeutic target in ovarian cancer, plays an important role in the development, progression, and immunotherapy of ovarian cancer and can be modulated by ubiquitination and deubiquitination modifications (Fig. 5B). However, few studies have combined p53 ubiquitination and deubiquitination with immunotherapy, and further experiments are needed to explore the possibility of combination use.

### CAR-T

**Figure 6:**
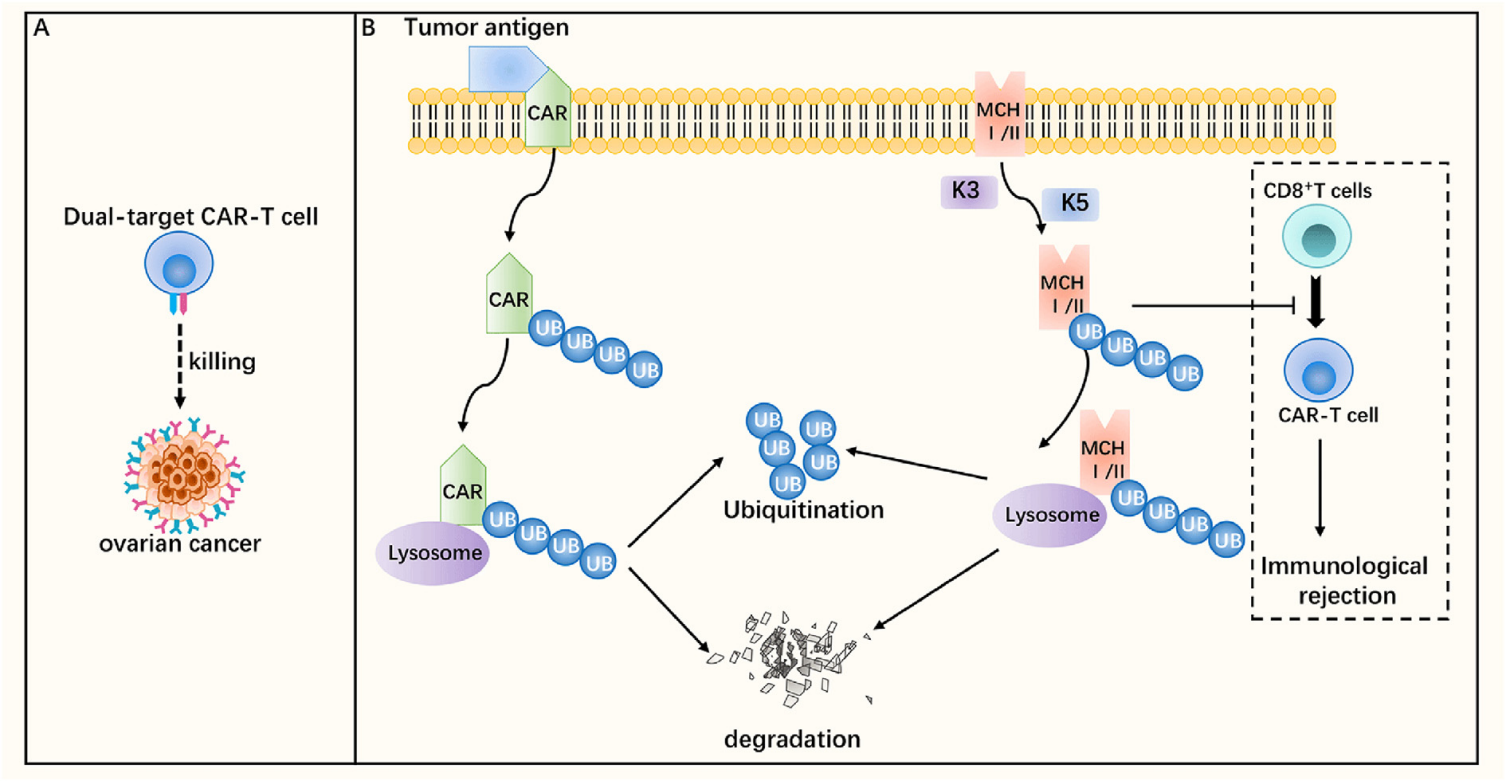
CAR-T cell immunotherapy with ubiquitination/deubiquitination. **(A)** Dual-targeted CAR-T cells have a strong anti-tumor activity to damage tumor cells. **(B)** The interaction between tumor antigen and CAR can promote the ubiquitination of CAR and its degradation by lysosomes. The ubiquitinase K3 and K5 proteins can promote the ubiquitination and lysosomal degradation of MCH I/II, thus blocking the recognition of CAR-T cells by the CD8^+^ T cells in the body, preventing the immune rejection reaction, and facilitating the survival of CAR-T cells for better therapeutic effects. CAR, chimeric antigen receptor.

Chimeric antigen receptor (CAR) T-cell therapy is a rapidly evolving therapeutic approach in adoptive cell transfer therapy, where artificial T cell receptors are generated by genetically engineering modified T cells. CAR is a recombinant receptor consisting of three components, an extracellular antigen recognition domain, an intracellular signaling domain involved in T-cell activation and killing, and a CD3ζ T-cell activation domain, which targets and destroys tumor cells. While CAR-T cell therapies have been highly successful in the treatment of hematological diseases, they are still under-researched for solid tumors, such as ovarian cancer.^101^^,^^102^ Firstly, CAR-T can directly target and kill tumor cells. It was found that mesothelin is highly expressed in ovarian cancer, and MSLN CAR-T cells can target and kill tumor cells with strong anti-tumor activity, and clinical reports showed that it has safety and efficacy.^103^^,^^104^ Due to the heterogeneity of ovarian cancer, single-target CAR-T cell therapy often leads to recurrence, so the study explored the efficacy of dual-target CAR-T cells. Several studies have shown that dual-target CAR-T cell therapy has stronger anti-tumor properties, such as MLSN constructing dual-target CAR-T cells with CD40,^105^ FOLR1 (folate receptor 1)^106^ and TAG-72 and CD47 dual-target CAR-T cells,^107^ which were found to have stronger anti-tumor performance than single-target CAR-T cells against cells with stronger killing ability (Fig. 6A). Secondly, CAR-T can regulate the tumor microenvironment and improve the ability to kill tumor cells. For example, for the regulation of tumor-associated macrophages and resident memory T cells, targeting the macrophage marker F4/80, CAR-T cells can target tumor-associated macrophages to suppress them and provide enhancement of tumor immunity,^108^ and CAR-T cells targeting CXCR6 can regulate resident memory T cells and thus modulate tumor immunity.^109^ Also, the combination of two CAR-T cells targeting tumor cells and targeting tumor microenvironment has been shown to improve the effectiveness of treatment and perform better anti-tumor function. Pretreatment with folate receptor beta-specific CAR-T cells prior to anti-mesothelin CAR-T cell therapy can modulate the tumor immune microenvironment and improve the effectiveness of anti-mesothelin CAR-T cells, which is conducive to better therapeutic effects.^110^

However, there are still many problems with CAR-T cell therapy, which has been found to be regulated by ubiquitination/deubiquitination, and ubiquitination/deubiquitination can improve the survival of CAR-T cells as well as the therapeutic effect. On the one hand, ubiquitination/deubiquitination can regulate the survival of CAR-T cells. Although off-the-shelf CAR-T cells can be produced in large quantities and used in multiple patients, the immune rejection caused by human leukocyte antigen (HLA) differences that affect CAR-T cell survival cannot be ignored. The E3 ubiquitin ligases K3 and K5 derived from human herpes virus-8 were found to ameliorate this immune rejection response due to the ubiquitous degradation of MHC I and II on CAR-T cells by K3 and K5, thereby evading the host immune response and maintaining the survival of off-the-shelf cells.^111^ Similarly, the E3 ubiquitin ligase Cbl-b can also regulate the survival of CAR-T cells, and CAR-T cells are resistant to endogenous depletion in the absence of Cbl-b, but the exact mechanism is not yet clear.^112^ On the other hand, ubiquitination regulation can also modulate CAR-T cell efficacy by affecting CAR signaling expression. Tumor antigen-stimulated rapid ubiquitination of CAR can lead to CAR lysosomal degradation, and when its ubiquitination is blocked, it can effectively enhance CAR signaling and improve the persistence of CAR-T cells with better tumor-killing ability.^113^ In addition to this, the E3 ubiquitin ligase UBR5 promotes the formation of immunosuppressive tumor microenvironment in ovarian cancer by regulating cytokines. The immunosuppressive microenvironment is improved when UBR5 is inhibited, resulting in improved therapeutic efficacy of CAR-T.^21^

Overall, the process of ubiquitination can modulate the efficacy of CAR-T cell therapy by regulating CAR signaling on CAR-T cells as well as the survival of CART cells, and thus ubiquitination is a potential target for tumor immunotherapy in combination with CAR-T (Fig. 6B). However, current mouse model studies do not fully mimic the *in vivo* conditions of cancer patients, and further clinical trials are needed for further exploration.

### TCR-T

TCR-T is similar to CAR-T and is an adoptive cell transfer therapy, but unlike the limited role of CAR-T in solid tumors, TCR-T has a better therapeutic effect in solid tumors.^114^^,^^115^ Engineered T-cell receptors (TCRs) are used to activate TCR-T cells to exert a killing effect by modifying TCRs to recognize tumor-expressed antigens. The reason why TCR-T has better efficacy in solid tumors is that TCR-T not only recognizes antigens on the surface of the tumor but also recognizes intracellular proteins presented by MHC, which greatly increases the pool of targeted antigens for TCR-T therapy.^116^ However, this also suggests that TCR-T cell therapy is MHC-restricted and relies on the presentation of MHC molecules to recognize targets and activate T-cell function, while loss of HLA-1 somatic cells in immunologically “cold” tumors also negatively affects the efficacy of TCR-T.^117^ Thus, CAR-T cells recognize surface antigens mainly outside the tumor, whereas TCR-T can penetrate the tumor and have a better therapeutic environment.^114^^,^^116^ In addition, TCR-T signal lasts longer than CAR-T, which is related to the fact that TCR itself exists in the body's immune system without causing immune rejection and immune memory of TCR-T.^114^^,^^117^

The benefits of TCR-T in immunotherapy for solid tumors are also seen in ovarian cancer. TCR-T has great therapeutic potential for ovarian cancers, especially highly aggressive cancers such as highly plasmacytoid cancers (high-grade serous ovarian cancer) and ovarian sarcomas (ovarian carcinosarcoma), which are often considered “cold” tumors.^117^ Targeting TCRs against the cancer assessment antigens NY-ESO1, MAGE-A4, and PRAME has been studied in clinical trials in patients with ovarian cancer.^117^ Recent studies have found that PRAME and CTCFL are highly expressed as tumor-specific tumor-associated antigens in ovarian cancer, and a series of TCR-T cells constructed such as PRAME TCR (DSK3^PRAME/QLL/A2^, 16.3C1^RAME/LYV/A24^ and 8.10C4^PRAME/SPS/B7^) and CTCFL TCR (39.2 E12^CTCFL/KLH/A2^) showed potent and specific anti-tumor responses *in vitro* and *in vivo*, showing promise as effective agents for ovarian cancer treatment.^118^ The absence of somatic HLA class I is a widespread mechanism of immune evasion, which affects the therapeutic efficacy of TCR-T since TCR-T recognition is dependent on the presentation of MCH molecules.^119^ A previous study showed that ubiquitination can regulate HLA-1 expression, but not in a direct way. P53 can up-regulate endoplasmic reticulum aminopeptidase 1 expression by binding to a homologous response element in the ERAP1 gene, resulting in increased HLA1 expression. MDM2 can regulate this process by degrading p53 through ubiquitination, and thus antagonizing this ubiquitination process can effectively increase the expression of HLA1, which may help to ensure the efficacy of TCR-T.^120^^,^^121^ Further research is needed to explore more effective treatment options.

### Wnt/β-catenin signaling pathway

**Figure 7:**
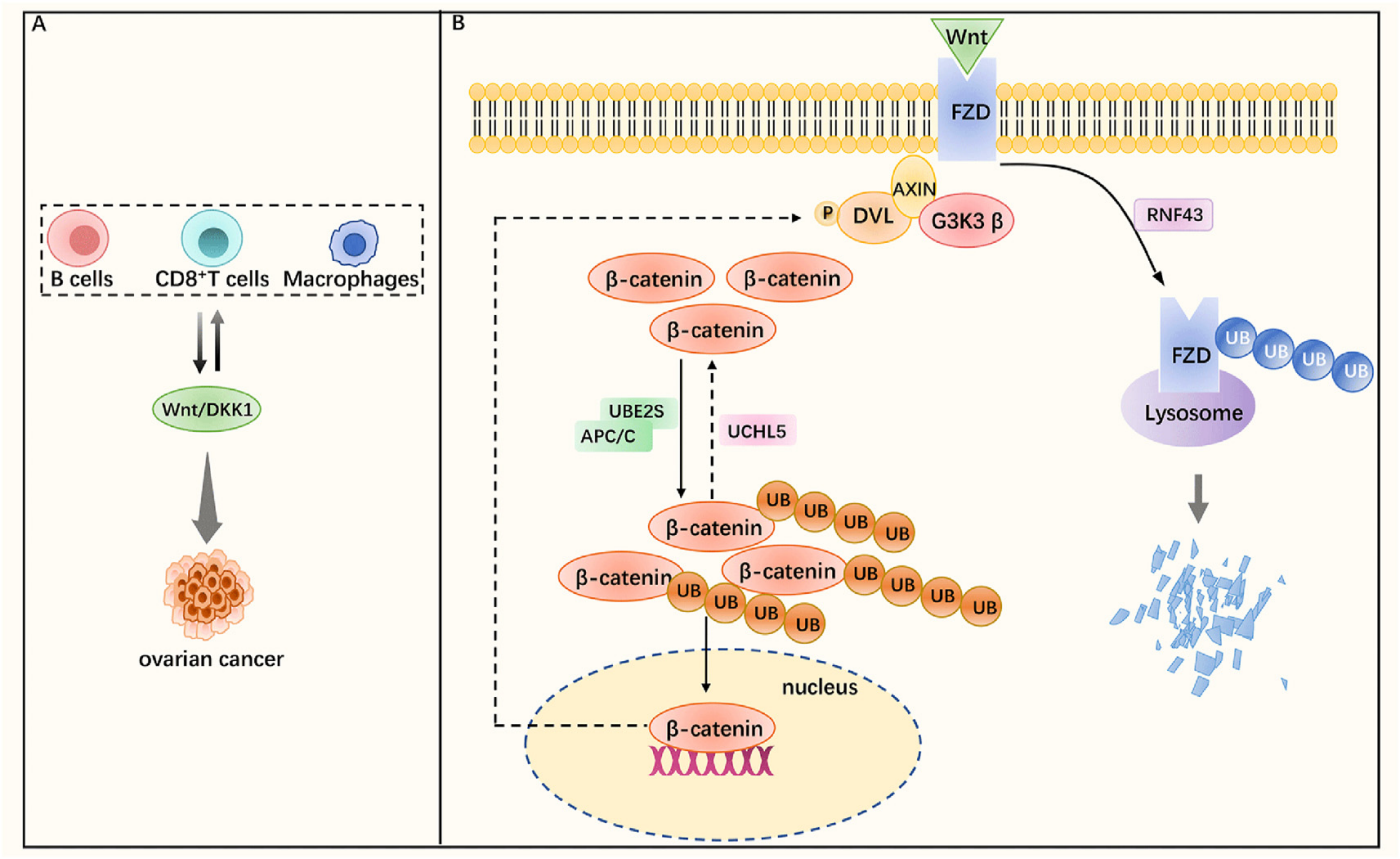
Wnt/β-catenin signaling pathway with ubiquitination/deubiquitination. **(A)** The Wnt signaling pathway regulates the infiltration of B cells, T cells, and macrophages in the tumor microenvironment, thereby affecting the anti-tumor immune response. **(B)** Wnt binds to the ligand FZD (Frizzled), which activates Dvl (Dishevelled) and recruits the “destruction complex” composed of AXIN and G3K3 β, thereby reducing the degradation of β-catenin and inducing β-catenin transfer to the nucleus. The ubiquitinase RNF43 catalyzes the ubiquitination and lysosomal degradation of FZD, thereby inhibiting the Wnt signaling pathway. The ubiquitinase UBE2S binds to APC/C to promote ubiquitination of β-catenin, enhance its stability, facilitate its translocation from the cytoplasm to the nucleus, and activate the Wnt signaling pathway. The deubiquitinase UCHL5 deubiquitinates β-catenin and reduces its degradation.

The Wnt/β-catenin signaling pathway is a highly evolutionarily conserved pathway that regulates key functions such as cell proliferation, differentiation, migration, genetic stability, apoptosis, and stem cell renewal.^122^ Wnt pathway has been shown to play a role in ovarian cancer development.^123^ Previous studies have found that the Wnt pathway is closely related to cancer cell growth and metastasis, and recently there is increasing evidence that the signaling of the Wnt pathway is related to the immunological regulation of the tumor microenvironment in ovarian cancer. One target in the Wnt/β-catenin signaling pathway is the ligand, Wnt itself, which controls the activity of the pathway, and the Wnt ligand is secreted from the cell after modification by PORCN enzymes to promote signal transduction.^124^ Wnt ligand binds to its two receptors Frizzled (FZD) and low-density-lipoprotein-associated receptors 5 and 6 and initiates intracellular signaling through β-catenin.^125^ AXIN and G3K3β form a “destruction complex” and activate the degradation of β-catenin. After Wnt binds to FZD, Dvl (Dishevelled) is activated and recruits the “destruction complex” to the cell membrane, thus inhibiting the degradation of β-catenin, inducing β-catenin activation and translocation to the nucleus, then activating the Wnt signaling pathway.^126^ Dickkopf-1 is a Wnt target gene that encodes a dickkopf-1 protein that competes with Wnt ligands for LRP5/6 binding and negatively regulates the Wnt pathway.^127^ The specific features of ovarian malignancies are poor tumor antigenicity and relative immune cell deficiency.^128^ Recent studies have found that β-catenin levels are associated with T cell rejection and tumor growth, that immune cell infiltration in the ovarian cancer tumor microenvironment can be regulated by modulating signal transduction in the Wnt pathway, that the PORCN inhibitor CGX-1321 increases infiltration of CD8^+^ T cells into the tumor microenvironment, and that DDK1 overexpresses myeloid-derived suppressor cells to promote immune evasion.^129^^,^^130^ Initially, the combination of CGX-1321 and DKN-01 (a DDK1 inhibitor) was not found to increase CD8^+^ T cell levels more significantly, but a subsequent study showed that DKN-01 increased HLA/MHC I expression in ovarian cancer tissues, which could increase their antigenicity and reduce immune escape. When treated with DKN-01 followed by CGX-1321, known as Sequential Wnt modulation, a variety of leukocytes are recruited into the tumor microenvironment by modulating Wnt signaling, including B cells and macrophages in addition to CD8^+^ T cells.^131^^,^^132^ In summary, the Wnt pathway can regulate the infiltration of multiple immune cells in the tumor microenvironment of ovarian cancer and has the potential to become a target axis for immunotherapy in oncology (Fig. 7A).

It was found that the Wnt signaling pathway in ovarian cancer can be regulated by ubiquitination/deubiquitination, including ubiquitinated beta proteins, FZD, and c-MYC proteins downstream of the Wnt pathway. The E3 ubiquitin ligase RNF43 can induce FZD ubiquitination and lysosomal degradation to negatively regulate the Wnt pathway, but RNF43 mutations are found in ovarian cancer, and its mutation frequency is second only to KRAS in mucinous ovarian cancer. Some of the mutations do not change their ability to negatively regulate the Wnt pathway, but the R117fs shift code mutation loses its negative regulation of the Wnt pathway to enhance the Wnt signaling pathway, and this induction of Wnt signaling pathway enhancement was inhibited by the Wnt inhibitor LGK974, suggesting that the Wnt pathway activity can be regulated by altering the E3 ubiquitin ligase.^125^^,^^133^ However, unlike the ubiquitinated degradation of FZD, β-catenin can be ubiquitinated to improve its stability and prevent its degradation.^134^ The E2 enzyme UBE2S is up-regulated in high-grade serous ovarian cancer, and the E2 enzyme UBE2S forms a complex with the E3 enzyme APC/C to promote the formation of a K11-linked ubiquitin chain from the k19 residue of the β-catenin, facilitating its accumulation in the cytoplasm and translocation to the nucleus, thereby activating the Wnt signaling pathway.^134^^,^^135^ In addition, the E3 ubiquitin ligases MARCH7 and MARCH1 and the DUB UCHL5 can also regulate β-catenin expression and thus the Wnt pathway, but the exact mechanism remains to be investigated.^136^, ^137^, ^138^ A recent study demonstrated that the E3 ubiquitin ligase TRIM37 binds to HUWEI and inhibits c-MYC protein expression, thereby affecting the Wnt pathway, but the exact mechanism needs to be investigated in depth.^139^

In conclusion, the Wnt pathway serves as a novel and effective pathway for regulating immune cell infiltration in the tumor microenvironment and can be regulated by ubiquitination and deubiquitination. Thus, the potential exists for ubiquitination and deubiquitination as a means of tumor immunotherapy (Fig. 7B). However, studies are not yet well established and further research is needed.

### Combined application of ubiquitinase/deubiquitinase inhibitors and other immunotherapeutic strategies

Protein post-translational modifications were found to play an important role in cancer development and have potential as cancer therapeutic targets.^140^ As key enzymes involved in the ubiquitination/deubiquitination process, E3 ubiquitin ligases and DUBs can both play a role in cancer immunotherapy, despite their opposing functions in regulating protein stability and potentially leading to different physiological responses. E3 ubiquitin ligases promote the process of ubiquitination, and the inhibitors of these enzymes can play a role in tumor immunotherapy by inhibiting the process.^141^^,^^142^ DUB inhibitors also play a role in combination with tumor immunotherapy by inhibiting the process of deubiquitination.^142^^,^^143^ Many inhibitors of E3 ubiquitin ligases and DUBs have been studied and found to affect the immune response against cancer cells, which in combination with other immunotherapeutic approaches can effectively reduce therapeutic resistance and improve therapeutic efficacy (Table 2).^70^^,^^144^

**Table 2:** Combined application of ubiquitinase/deubiquitinase inhibitors and immune checkpoint inhibitors/cancer vaccines/anti-tumor antibodies.

Combined application	Ubiquitin/deubiquitinating enzyme	Inhibitors	Combined immune checkpoint inhibitors	Phases	Cancer types	Effects	Reference
Immune checkpoint inhibitors	cIAP1/2, XIAP	ASTX660	PD-1 antibody XRT	Pre-clinical	Head and neck squamous cell carcinoma	It promotes the sensitivity of tumor cells to TNF-α and enhances the killing effect of T cells on tumor cells.	155
	IAP	Birinapant	PD-1 antibody	Pre-clinical	Colon cancer	It makes tumor cells more sensitive to TNF-mediated killing, and the combination with PD-L1 inhibitors further enhances the killing ability of tumor cells.	156
	cIAP1/2	LCL161	PD-1 antibody/cytotoxic T-lymphocyte antigen 4 antibody	Pre-clinical	Glioblastoma	It sensitizes tumors to TNF-α-induced killing, enhances the activity of cytotoxic T cells against tumors, and enhances the effect of immune checkpoint inhibitors in combination with immune checkpoint inhibition therapy.	158
	cIAP1/2	LCL161	PD-1 antibody	Pre-clinical	Multiple myeloma	It can modulate the tumor microenvironment and induce powerful immune activation, thus stimulating anti-tumor phagocytic activity and durable anti-tumor immunity.	157
	MDM2	APG-115	PD-1 antibody	Pre-clinical	Liver cancer, colon adenocarcinoma	It inhibits ubiquitinated degradation of p53 caused by MDM2, regulates the infiltration of immune cells in the tumor microenvironment, and enhances anti-PD-1 efficacy.	148
	MDM2	AMG-232	Pembrolizumab	Pre-clinical	Ovarian clear cell carcinoma	It targets MDM2 and enhances T cell-mediated tumor killing.	69
	MDM2	HDM201	Anti-PD-1 antibody, anti-PD-L1 antibody	Pre-clinical	Colon cancer	It inhibits the interaction between MDM2 and P53, increases dendritic cells, up-regulates CD8^+^/Treg ratio, and promotes durable tumor-specific immune responses to improve anti-PD-1/PD-L1 immune responses.	160
	USP7	P5091	PD-1 antibody	Pre-clinical	Lung cancer	It inhibits the activity of Treg and preserves the function of T cells as effector cells to promote anti-tumor immunity.	161
Cancer vaccines	USP7	P5091	Adenovirus vaccines	Pre-clinical	Lung cancer	It inhibits the activity of Treg and preserves the effector cell function of T cells to promote anti-tumor immunity.	161
	IAP	M1	B16 vaccines (GVAX)	Pre-clinical	Melanoma	It enhances the response of CD4^+^ CD8^+^ T cells, and other anti-tumor cells, which can be used in combination with tumor vaccines to enhance the therapeutic effect.	166
	MDM2	Nutlin-3	MDM2_32-46_ peptide vaccine	Pre-clinical	Head and neck squamous cell carcinoma	It blocks the interaction of MDM2 with p53, up-regulates the expression of tumor HLA to enhance the anti-tumor response of MDM2-specific T cells, and promotes vaccine-induced T cell killing of tumors.	120
	IAP2, XIAP	Smac mimetic	BCG	Pre-clinical	Bladder cancer	It promotes the killing of tumor cells by acting through TNF-α secreted by BCG-stimulated neutrophils.	167
Anti-tumor antibody	USP1	Pimozide	Rituximab	Pre-clinical	Large B-cell lymphoma	It inhibits the USP1-mediated deubiquitination of MAX/MYC protein, which helps to reduce the resistance to rituximab treatment and inhibits the proliferation of rituximab-resistant cells.	179
CAR-T	IAP	Birinapant	CAR-T	Pre-clinical	Breast cancer	It sensitizes tumor cells to CAR-T cell-derived TNF, significantly enhancing the antitumor activity of CAR-T lymphocyte therapy *in vitro* and *in vivo*.	169

### Combined application of ubiquitinase/deubiquitinase inhibitors and ICBs

Today's cancer immunotherapy has become an effective cancer treatment, with ICBs approved for the treatment of multiple different cancers, the more common being ICB against cytotoxic T-lymphocyte antigen 4 or PD-1-PD-L1 axis.^145^ Although ICBs have been used to treat a variety of cancers, most patients still do not show an effective therapeutic response against ICBs, which may be related to local immunosuppression of the tumor.^146^ Inhibitors of ubiquitinase/deubiquitinase can modulate the tumor microenvironment and improve the sensitivity of tumors to T-cell killing, and their combination with ICB has been shown to significantly improve the therapeutic efficacy of ICB.^147^^,^^148^ Therefore, the combination of drugs can improve ICB drug resistance and enhance the efficacy of tumor treatment.

Firstly, E3 ubiquitin ligase inhibitors may improve the efficacy of ICB therapy. Inhibitory apoptosis proteins (IAPs) are a group of E3 ubiquitin ligases that include eight members: cIAP1, cIAP2, NAIP, Survivin, XIAP, Bruce, ILP-2, and Livin.^149^ Abnormally high expression of IAPs is a common oncogenic event in human cancers, and IAPs block cystatinase-mediated apoptosis by binding to and ubiquitinating degraded cystatinases.^149^, ^150^, ^151^ Various IAP inhibitors such as LCL161, Debio1143, and birinapant have been developed and found to be effective in inhibiting cancer.^152^, ^153^, ^154^ The IAP inhibitor ASTX660 sensitized a mouse model of head and neck squamous cell carcinoma to tumor necrosis factor alpha (TNF-α) and sensitized tumor cells to perforin/granzyme B, TNF-α, TRAIL, and FasL-mediated antigen-specific T-cell killing, but did not damage the host, and its combination with PD-1 antibody showed enhanced antitumor activity, thereby significantly delaying or eradicate tumors.^155^ The IAP inhibitor birinapant makes tumor cells more sensitive to CL-derived TNF killing, while TNF is elevated after PD-1 blockade, and the combination of the two further enhances the efficacy of anti-PD-1 antibody therapy.^156^ Another IAP inhibitor, LCL161, modulates the tumor microenvironment of multiple myeloma and induces strong immune activation, thereby stimulating anti-tumor phagocytic activity and durable anti-tumor immunity; when combined with LCL161 and PD-1 antibody, it significantly enhances anti-PD-1 antibody efficacy.^157^ In addition to enhancing the efficacy of anti-PD-1 therapy, IAP inhibitors can also be combined with anti-cytotoxic T-lymphocyte antigen 4 to induce tumor cell sensitivity to killing. LCL161 sensitizes tumors to TNF-α-induced killing, enhances cytotoxic T lymphocyte activity against tumors, and promotes therapeutic efficacy in glioblastoma in combination with anti-cytotoxic T-lymphocyte antigen 4, and similar results have been found in other cancers such as breast cancer.^158^ MDM2 is one of the E3 ubiquitin ligases that binds to the tumor suppressor p53 and mediates the ubiquitinated degradation of p53, and overexpression of MDM2 is a factor in the poor prognosis of various tumors.^120^^,^^159^ In recent years, several MDM2 inhibitors have been found that can be combined with ICB to improve the tumor treatment effect of ICB. Instead of working by degrading MDM2, these inhibitors inhibit MDM2-mediated p53 ubiquitination by blocking the binding of MDM2 to p53.^69^^,^^148^ AMG232 is an investigational orally bioavailable selective MDM2 inhibitor, and the combination of AMG232 and pembrolizumab anti-PD-1 treatment reduces IL-6 expression as well as enhances T cell-mediated tumor killing in ovarian cancer cell lines.^69^ APG-115 inhibits the interaction between MDM2 and p53, which promotes the activation of p53 in tumor microenvironment immune cells, resulting in increased infiltration of CD8^+^ T cells and M1 macrophages while reduced infiltration of M2 macrophages, enhancing the anti-PD-1 antibody anti-tumor immune efficacy.^148^ Similarly, HDM201, an MDM2 inhibitor, inhibited the ubiquitinated degradation of p53 by MDM2, increased dendritic cells as well as the CD8^+^/Treg ratio, promoted a durable tumor-specific immune memory response and improved the anti-tumor response of anti-PD-L1 antibody/anti-PD-1 antibody against colon cancer.^160^ In addition to E3 ubiquitin ligases, deubiquitinases can also influence the efficacy of ICB therapy. USP7 is one of the most studied deubiquitinases and plays a critical role in mediating MDM2 stabilization, and the combination of USP7 inhibitors with ICB was also found to improve therapeutic efficacy. P5091 is a USP7 inhibitor that inhibits Treg activity and preserves key T effector cell function in a mouse lung cancer model. P5091 promotes anti-tumor immunity and its activity is significantly increased in combination with anti-PD-1 antibodies.^161^

There have been several clinical trials on the combination of ubiquitinase and ICB, further improving the understanding of their combined use. A clinical Ib study in patients with metastatic solid tumors found that APG-115 in combination with pembrolizumab was well tolerated and its promising anti-tumor effects have been seen in several tumor types.^162^ Meanwhile, most clinical trials are still ongoing, such as APG-115 in patients with advanced solid tumors or lymphoma (NCT02935907), PDR001 in combination with LCL161, everolimus, or panobinostat (NCT02890069), multiple immunotherapy-based therapeutic combinations in patients with metastatic colorectal cancer (Morpheus-CRC) (NCT03555149), atezolizumab and cobimetinib or idasanutlin in participants with stage IV or unresectable recurrent estrogen receptor positive breast cancer (NCT03566485), and birinapant and pembrolizumab in solid tumors in a dose escalation study (NCT02587962). The subsequent results of these studies will be beneficial in increasing our knowledge of the combination. Therefore, the combination of ubiquitinase/deubiquitinase and ICB is a promising way to improve treatment resistance and enhance the efficacy of tumor immunotherapy.

### Combined application of ubiquitinase/deubiquitinase inhibitors and cancer vaccines

Prophylactic and therapeutic vaccines are representative strategies for cancer immunotherapy. The former aims to induce immune memory by administering vaccines to healthy individuals to prevent the onset of disease caused by specific cancers. The latter is used for disease management in cancer patients by boosting or activating the patient's own immune system. Cancer vaccines can be classified into three main categories based on the technology and content they use, namely cellular vaccines (tumor or immune cells), protein/peptide vaccines, and nucleic acid vaccines (DNA, RNA, or viral vectors).^163^ Until now, the most used vaccine is a preventive vaccine against human papillomavirus-related cervical cancer.^164^ Currently, except for some specific cancer vaccines, monotherapies using cancer vaccines usually have only minimal clinical effect. The lower efficacy of monotherapy may be caused by multiple immune evasion mechanisms in cancer, with the immunosuppressive tumor microenvironment playing an important role.^163^^,^^165^

For E3 ubiquitin ligase inhibitors, several studies have demonstrated that they can influence tumor vaccine therapy. The MDM2 inhibitor Nutlin-3 can block the interaction between MDM2 and p53. This maintains the stability of p53 thereby increasing tumor HLA class I expression via endoplasmic reticulum aminopeptidase 1^121^ up-regulation of tumor HLA expression and enhance the anti-tumor response of MDM2-specific T cells, which can effectively promote MDM2_32-46_ peptide vaccine-induced T cell killing of tumor cells.^120^ In addition, the IAP inhibitor M1 enhances the response of CD4^+^ CD8^+^ T cells, and other anti-tumor effector cells and the combination with B16 vaccines (GVAX) improves the efficacy of tumor vaccines.^166^ Smac mimetic, an inhibitor of IAP2 and XIAP, acts through TNF-α secreted by BCG-stimulated neutrophils to promote BCG-induced killing of tumor cells, and the combination of the two drugs effectively promotes the treatment of bladder cancer.^167^ For deubiquitinating enzyme inhibitors, in a study of a mouse lung cancer model, it was found that the combination of USP7 inhibitor P5091 and adenovirus vaccine significantly increased infiltration of tumor-specific CD8^+^ T cells and IFN-γ production with reduced accumulation of Foxp3^+^ Treg cells in the tumor, resulting in a significant limitation of tumor growth.^161^ In conclusion, the combination of tumor vaccines with ubiquitinase/deubiquitinase inhibitors has a greater therapeutic potential, but more studies are still needed to determine the specific mechanisms and effects.

### Combined application of ubiquitinase/deubiquitinase inhibitors and CAR-T

CAR-T cell therapies have been proven successful in treating hematologic malignancies, particularly acute lymphoblastic leukemia and B-cell lymphoma, and the FDA has approved five CAR-T therapies for hematologic malignancies.^168^ However, the poor efficacy of CAR-T cells in solid tumors may be due to tumor-associated immunosuppression. Some of the major barriers to CAR immunotherapy in solid tumors include CAR-T cell manufacturing, lack of tumor-specific antigens, inefficient CAR-T lymphocyte transport and infiltration of tumor sites, immunosuppressive tumor microenvironment, treatment-related toxicity, and antigen escape.^168^^,^^169^ As an antagonist of IAP, birinapant inhibits IAP to sensitize tumor cells to CAR-T cell-derived TNF and significantly enhances the anti-tumor activity of CAR-T lymphocyte therapy *in vitro* and *in vivo*. Combining CAR-T cell therapy with birinapant significantly inhibits tumor growth in mice *in vivo*, with stronger therapeutic effects than treatment alone.^169^ CAR-T therapy is less durable because CARs are susceptible to degradation by ubiquitination. It was found that the ubiquitination of CAR could be blocked by mutating all cytoplasmic lysine to arginine, and such cells were called retrievable CAR-T cells. Such recyclable CAR-T cells have been shown to have better long-term killing capacity, better durability, and enhanced anti-tumor capabilities.^113^

In conclusion, studies have shown that the modulation of the ubiquitination pathway in combination with CAR-T cells can improve the efficacy of solid malignant tumor overt cell therapy, providing a new idea for improving CAR-T cell therapy.

### Combined application of ubiquitinase/deubiquitinase inhibitors and anti-tumor antibodies

Nowadays, the immunotherapy of cancer is rapidly developing, among which anti-tumor antibodies are a therapeutic strategy that cannot be ignored. Monoclonal antibodies for tumor treatment are mainly based on three mechanisms: (i) the inhibition of factors and receptors that activate signaling pathways in cancer cell division and angiogenesis through antibody binding; (ii) the antibody-dependent cellular cytotoxicity (ADCC) such as rituximab, transtuzumab, cetuximab, and pertuzumab; (iii) complement-dependent cytotoxicity such as rituximab, alemtuzumab, cetuximab, and ofatumumab.^170^, ^171^, ^172^ The main types of cancers targeted by monoclonal antibodies are breast cancer, colon cancer, lymphoma, *etc*.^173^

ADCC is a key mechanism for the anti-tumor effects of clinically applied anti-tumor antibodies. It was found that the ADCC therapeutic effect of anti-tumor monoclonal antibodies was facilitated by increasing the number and activity of natural killer cells.^174^ It is possible that the ubiquitin-proteasome pathway could have an influence on ADCC by regulating natural killer cells, which in turn could improve therapeutic efficacy. The E3 ubiquitin ligase CRBN complex facilitates the ubiquitination degradation of IKZF1/3, thereby rescuing the inhibition of IKZF1/3 on natural killer cells. Well, this process can be facilitated by the immunomodulatory drug pomalidomide and enhances the ADCC effect.^175^ Thus, the ubiquitinated proteasome pathway may serve as a potential target in modulating ADCC effects and promoting the efficacy of anti-tumor monoclonal antibody therapy. Also, the NF-κB signaling pathway plays an important role in tumor immunity and is also closely related to the efficacy of monoclonal antibody therapy.^74^^,^^176^ The ubiquitinated proteasome pathway can influence the therapeutic efficacy of monoclonal antibodies by regulating NF-κB. Trastuzumab is a commonly used anti-tumor monoclonal antibody, but the immunosuppressive microenvironment of tumors promotes drug resistance to trastuzumab and hampers therapeutic efficacy. TRAF6/3 acts as an E3 ubiquitin ligase and induces its proteasomal degradation through k48-linked self-ubiquitination, thereby inhibiting NF-κB signaling and promoting drug resistance in tumors. Whereas targeting CD40 inhibits ubiquitination of TRAF6/3, and drug resistance is overcome by treatment with anti-CD40-scFv-linked anti-HER2 (CD40 × HER2) bispecific antibody (bsAb).^177^ In addition to this, brentuximab vedotin (BV), a drug-coupled anti-CD30 antibody, is one of the most effective therapeutic agents for patients with refractory/recurrent Hodgkin's lymphoma, but there are still many instances of therapy resistance to brentuximab vedotin. Ubiquitin editing enzyme A20 down-regulates TNF-α-induced NF-κB signaling by catalyzing ubiquitination of receptor-interacting protein and negatively regulates the activity of NF-κB, and thus up-regulates brentuximab vedotin sensitivity.^176^^,^^178^

A recent study found that the USP1 inhibitor pimozide inhibited the deubiquitination of MAX/MYC proteins by USP1, induced apoptosis, autophagy, and cell cycle arrest in tumor cells, and improved rituximab treatment of diffuse large B-cell lymphoma resistance.^179^ This provides new insights into the use of ubiquitinase/deubiquitinase inhibitors in combination with anti-tumor antibodies, suggesting that this may be another way to improve the efficacy of tumor therapy.

## Conclusion

Ovarian cancer is one of the deadliest gynecologic malignancies in the world, with a 5-year survival rate of less than 50%, and despite multiple treatment options today, it has not been effective in improving overall survival rates.^180^ Ubiquitination and deubiquitination are important post-translational modification mechanisms that regulate the tumor microenvironment and host immune response to tumor cells, and they also play a key role in regulating the proliferation, migration, and invasion of ovarian cancer cells.^18^^,^^180^ In addition, other post-translational modifications such as phosphorylation and acetylation were found to regulate ovarian carcinogenesis together with ubiquitination/deubiquitination. For example, Gα13 is involved in cell proliferation, migration, and invasion, regulates large tumor suppressor kinase phosphorylation at serine 909 upon activation, and induces the recruitment of the itchy E3 ubiquitin ligase to trigger large tumor suppressor kinase 1 degradation. Large tumor suppressor kinase 1 is a key component of the Hippo signaling pathway, and its down-regulation promotes epithelial–mesenchymal transition in ovarian cancer epithelial cells, resulting in enhanced invasiveness.^181^ Lysine acetyltransferase 6 A is a MYST-type histone acetyltransferase. It binds to and acetylates COP1 at K294, which impairs the function of COP1 as an E3 ubiquitin ligase and leads to the accumulation and enhanced activity of β-catenin, thereby promoting the proliferation and migratory ability of ovarian cancer cells.^182^ Therefore, the combination of multiple post-translational modifications and ubiquitination/deubiquitination may be a potential new target for ovarian cancer therapy.

Studies have shown that ubiquitinase/deubiquitinase can be involved in cancer therapy as a target, and most studies have focused on IAP and MDM2 inhibitors, for example, the IAP inhibitor birinapant has been shown to be well tolerated in clinical trials with stable anti-tumor activity in some patients with solid tumors.^162^ Another phase I clinical trial also demonstrated that the MDM2 inhibitor ALRN-6924 was well tolerated and showed anti-tumor activity in patients with solid tumors and lymphomas carrying TP53.^183^ Further studies have found that ubiquitinase/deubiquitinase inhibitors can be used in combination with a variety of known immunotherapies to improve efficacy, including ICB, CAR-T cell therapy, tumor vaccines, and anti-tumor antibodies.^184^ Of these, anti-PD-1 therapy is the most intensively studied, with several clinical trials already underway.^148^^,^^160^

Although there is extensive research on cancer immunotherapy, on the one hand, the use of ubiquitinase/deubiquitinase as a target in combination with classical immunotherapeutic approaches in ovarian cancer is still inadequate, and on the other hand, there is a lack of sufficient clinical trials to drive research progress. In conclusion, research on the combination of ubiquitinase/deubiquitinase inhibitors with immunotherapy may provide more possibilities for improving the treatment of ovarian cancer in the future.

## Author contributions

Ting Sun and Liwei Ma conceived the structure of the manuscript and revised the manuscript. Huiling Guo and Jianwei Wei collected the related paper and drafted the manuscript. Yuyan Zhang and Junhu Wan created the figures. Weiwei Wang, Ling Gao, and Jiajing Li revised this manuscript. All authors read and approved the final manuscript.

## Conflict of interests

These authors declare that they have no competing interests.

## Funding

This project was supported by the 10.13039/501100001809National Natural Science Foundation of China (No. 82002751), the Medical Science and Technology Project of Henan Province, China (No. SBGJ202102139), 10.13039/501100002858China Postdoctoral Science Foundation (No. 2020M682361), Excellent Youth Foundation of Henan Province, China (No. 222300420071), Outstanding Young Talents of Health Science and Technology Innovation of Henan Province, China (No. YXKC2022033), and the Funding for Scientific Research and Innovation Team of The First Affiliated Hospital of Zhengzhou University, Henan, China (No. QNCXTD2023005).
